# Bibliometric analysis of traditional Chinese medicine for smoking cessation

**DOI:** 10.18332/tid/154961

**Published:** 2022-11-09

**Authors:** Jingli Xing, Jianping Liu, Mei Han, Yue Jiang, Jiali Jiang, He Huang

**Affiliations:** 1 Centre for Evidence-Based Chinese Medicine, Beijing University of Chinese Medicine, Chaoyang, China; 2 School of Economics and Management, China University of Geosciences, Beijing, China

**Keywords:** smoking cessation, therapy, TCM, bibliometric analysis

## Abstract

**INTRODUCTION:**

Smoking cessation is an efficient approach to reducing disease burden. Traditional Chinese Medicine (TCM) therapies such as acupuncture, acupressure, and herbal drugs are often used to help quit smoking. However, there is a lack of overarching bibliometric analysis of the clinical research on smoking cessation focusing on TCM. The aim of our study is to explore the current patterns and trends of TCM therapy for smoking cessation through bibliometric methods with visual presentation.

**METHODS:**

This study is an assessment of academic publications retrieved from the Scopus database on smoking cessation using TCM therapy published in the period 2005–2021. Sankey diagram, word-cloud, network analysis, thematic maps, tree-maps, and the collaborative work of authors, institutions and countries, were used to identify research trends on TCM therapy for smoking cessation. The total cited index and H-index (for journals, authors, countries, organizations) were used to identify the trends of worldwide development by R Package and Excel 2016.

**RESULTS:**

There was an upward trend, with some fluctuations, of 1908 articles from 2005 to 2021. The most productive country was China. The top institution in this field was Beijing University. The dominant author that contributed to TCM therapy for smoking cessation was Wang Y, who has the highest H-Index. The most productive cited journals were *Evidence-Based Complementary and Alternative Medicines and the Chinese Journal of Clinical Rehabilitation.* Liu L, (2011, STROKE) had the highest centrality. The keywords ‘acupuncture’, ‘traditional Chinese medicine’, ‘colitis’, ‘hypertension’, ‘chronic obstructive pulmonary disease’, ‘risk factors’ and ‘alternative medicine’ ranked highest in frequency. The diseases of healthy people concerned mainly cardiovascular, cancer, diabetes, hypertension and pregnancy. The diseases of the patients concerned mainly cancer, diabetes, hematopathy, stroke, cardiovascular, diabetes, lung disease, and hypertension. Treatment methods were mainly traditional Chinese medicine and acupuncture. The research methods mainly included randomized controlled trials that were multi-center and double-blind.

**CONCLUSIONS:**

A substantial number of articles on TCM therapy for smoking cessation, mainly focusing on TCM and acupuncture were identified. It is worth noting that research that focused on TCM therapy for smoking cessation also was related to COVID-19.

## INTRODUCTION

Tobacco dependence ranks first among the public health threats in the world today. On average, nearly 6 million people die each year of diseases caused by tobacco dependence. There are more than 5 million smokers, and more than 0.6 million people who do not smoke but often come into contact with smokers. There are several advantages of Traditional Chinese Medicine (TCM) therapy for smoking cessation, these are: fewer side effects, more operability, and can be adjusted according to an individualized therapy plan. As a common chronic disease, tobacco dependence is more common in long-term smokers. According to the China Adult Tobacco Survey Report in 2018, the percentage of people aged ≥15 years who smoke in China was 26.6%, of which males were 50.5%, females were 2.1%, the rate in rural areas was 28.9%, and the rate in urban areas was 25.1%. Smoking is one of the risk factors for many diseases such as chronic bronchitis, lung cancer, and pulmonary fibrosis^1^. The awareness of such diseases may be limited among adult smokers in China, and it may thus become a high-profile issue in the future. Existing studies have explored the mechanism and effectiveness of TCM therapy for smoking cessation^2^. First, clinical trials demonstrate the effectiveness of acupuncture for smoking cessation^3^. Second, several systematic reviews suggest that clinical application in combination with pharmacological intervention is useful^4^. In addition, basic research shows that smoking increases the risk of lung cancer^5^, cardiovascular disease, and chronic obstructive pulmonary disease in adults^6^. In view of the increasing number of published studies and the increasing diversity of topics, it is necessary to identify the efficacy of TCM for smoking cessation^7^ and to understand its current status.

Bibliometrics is a comprehensive analysis method that uses mathematical and statistical tools to assess the relationships and linkages among publications, journals, countries, and researchers within a research field^8^. Bibliometrics can be used to provide an overview of a large number of scholarly articles and to systematically estimate patterns of research trends. Furthermore, it can differentiate the influence of different countries, organizations, authors, publications and journals in a specific research field^9^. Bibliometric analyses have been used to assess research trends in certain areas, including complementary and alternative medicines. For example, bibliometric studies have been used to evaluate smoking cessation studies over the past few years^10^. Although bibliometric methods have been applied in many research areas, no detailed bibliometric analysis has been performed on the topic of TCM smoking cessation treatment. There is no detailed bibliometric and machine learning visualization analysis on TCM smoking cessation treatment.

The purpose of this study is to provide an overall analysis of current TCM smoking cessation therapies, to highlight trends and patterns, to explore changes in journals, institutions and author networks, and to investigate the structure and development of TCM therapies for smoking cessation. Therefore, the study used several bibliometric methods to present the following questions:

- Q1 – What are the research progress and trends of TCM for smoking cessation, and what issues are most worthy of discussion in this work?
- Q2 – Which are the most influential journals, the most productive authors and the leading articles in the research area?
- Q3 – Which are the collaborative networks that outline the conceptual structure of the field and the intellectual structure of the research community?
  Q3a – What are the ideas and methods of traditional Chinese medicine for preventing and treating tobacco dependence? Q3b – What reporting frameworks and formats were used?
- Q4 – What are the main disease and treatment methods in the study of TCM therapy for smoking cessation for healthy and patient populations?

## METHODS

### Literature search

Publications were retrieved by using computerized searches from Scopus, on 1 January 2022.

At the start of the study, we searched for all articles related to Chinese medicine for smoking cessation in Scopus from 2005 to 2021, to identify clear trends in changes that meet the requirements of bibliometric analysis.

**Figure 1:**
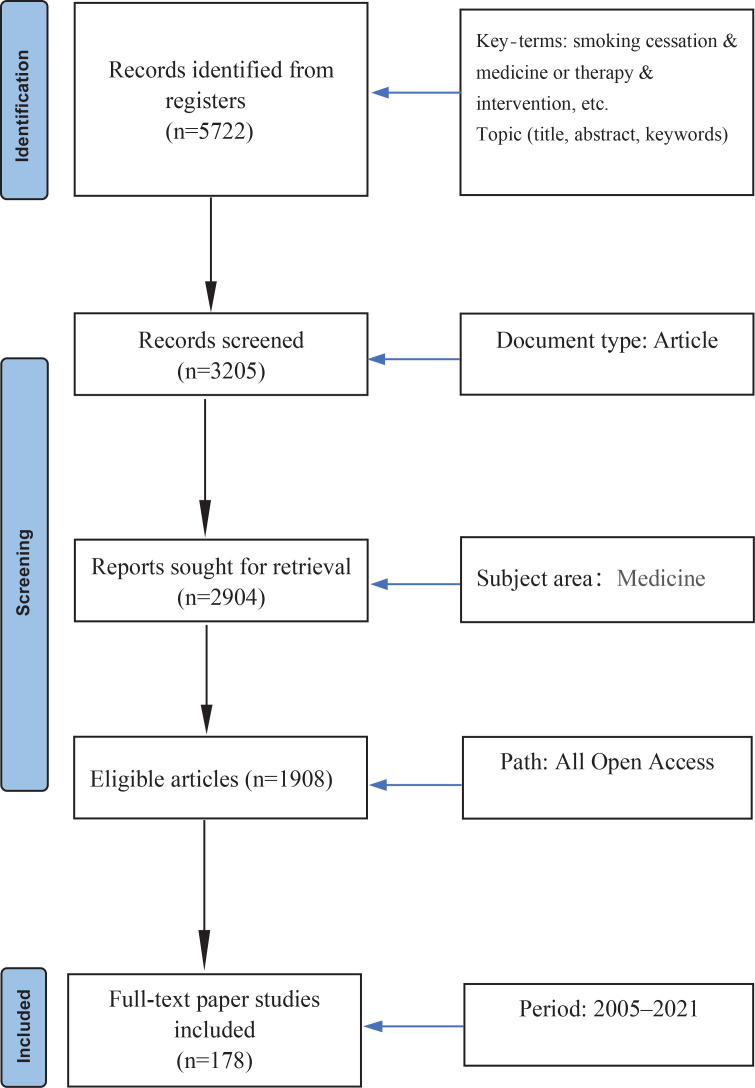
Study selection of TCM therapy for smoking cessation

The search strategy was as follows: [Title (smoking cessation)] AND [Title-ABS-Key (‘TCM’) OR Title-ABS-Key (random)] AND [limit-to (OA, ‘all’)] AND [limit-to (‘ar’, doctype)] AND [limit-to (‘medi’, subjarea)] AND [limit-to (‘human’, exactkeyword)] OR limit-to (‘smoking’, exactkeyword)]. After deduplication, 5042 articles were filtered by title, keywords and abstract by Excel 2011 and R software *biblioshiny*. Finally, 178 full-text papers were evaluated for eligibility (Figure 1).

The visualizations and distribution diagrams were established using R software *biblioshiny*, a statistical package, including data analysis and data visualization, which are available on R-Studio that facilitates comprehensive bibliometric research. The software is used to retrieve relevant bibliometric qualitative and quantitative information, such as a Sankey diagram, word-cloud, network analysis, thematic maps, tree-map, networks etc.

### Analysis methods

The methods of analysis not only included examining selected documents against the basic characteristics of the database (e.g. authors, journals, papers), but also included the identification of knowledge concepts and knowledge structures through visual methods (e.g. word-cloud, thematic maps, tree-map, three-field maps and network analysis). There was a geographical file generated by R *biblioshiny*, in order to analyze the geographical heatmap and collaboration network. The data were from Scopus, which is cleaned with Excel 2011 in the report. The total citation index (TC) and H-index were used to judge the dynamic development of the authors’ country. There were four stages of analysis from Q1 to Q4. The bibliometric tools used at each analysis stage and their purpose are listed in Table 1.

**Table 1:** The bibliometric tools used in each stage of the bibliometric analysis

*Stages*	*Description*
**First stage – growth trends (answering Q1)**	
Geographical distribution collaboration	
**Second stage – leading articles (answering Q2)**	
Leading journals	Description of research trends by the total cited index and H-index
Adding prolific authors	
Top cited papers	
Three-field plot	The visualization of selected fields by a Sankey diagram
**Third stage – 3C network analysis (co-occurrence, co-keywords, co-citation) (answering Q3 and Q4)**	
Tree-map	The tree-map explores the frequency and proportion of each word in the target topic, and presents the visual proportion in the size of the ‘rectangular’ area
Word-cloud	Display word frequency by size or thickness of keywords
Co-occurrence network	Visualization of research hotspots due to interactions between topics in research paper content
Thematic map	The group research topics are divided into four quadrants, expressing their development and relevance to areas of knowledge
Topic dendrogram	Description of the relationship between keywords through cluster analysis to create the conceptual structure

## RESULTS

### The overview of the information of sampled data

The analyzed sample included at least one in 178 scientific papers published in 1908 journal articles from 2005 to 2021. It revealed the size, growth trend and distribution of TCM therapy for smoking cessation over the period. The total number of articles had continued to increase over the past few years, with some fluctuations, peaking in 2020 with 31 articles published. Our bibliometric analysis of research showed that the total number of published papers on smoking cessation in traditional Chinese medicine had grown steadily from 2005 to 2021.

The overview information of articles about TCM therapy for smoking cessation literature is presented in Table 2. The sample data showed that most of the contribution came from mainland China (52.14%), USA (18.57%) and Japan (3.57%), according to the sample studied. Chinese scholars contributed significantly to the topic articles (73), and ranked first in terms of total citations (1368). Moreover, Canada had the highest average article citation (81.5).

**Table 2:** TCM related output and its citations of articles in different regions

*Region*	*Articles*	*Total citations*	*Average article citations*
China (mainland)	73	1368	18.7
USA	26	1133	43.6
China Hong Kong	8	89	11.1
Japan	5	88	17.6
Australia	4	189	47.2
United Kingdom	4	144	36
Malaysia	3	32	10.7
Singapore	3	48	16
Canada	2	163	81.5
Germany	2	148	74
Brunei	1	7	7
France	1	15	15
Greece	1	3	3
Israel	1	33	33
South Korea	1	9	9
Lebanon	1	37	37
New Zealand	1	15	15
Croatia	1	0	0
Brazil	1	0	0
India	1	0	0

The top two academic affiliations were Beijing University and Henan University of Traditional Chinese Medicine, presented in Table 3. The data showed that the majority of contributions were made by mainland China (77.96%), Hong Kong (10.17%), Singapore (4.24%), and India (4.24%), according to the sample studied.

**Table 3:** Output of articles by study affiliation

*Affiliations*	*Articles*	Location*
Beijing University	17	China
Henan University of Traditional Chinese Medicine	12	China
Capital Medical University	10	China
Huazhong University of Science and Technology	10	China
Naval Medical University (Second Military Medical University)	7	China
Hong Kong University	7	Hong Kong
China Academy of Chinese Medical Sciences	6	China
China Medical University	6	China
Fudan University	6	China
Central South University	5	China
Chinese University of Hong Kong	5	Hong Kong
National University of Singapore	5	Singapore
Sichuan University	5	China
Tata Memorial Center	5	India
Affiliated Hospital of China Medical University	4	China
Dalian Medical University	4	China
National Yang-Ming University	4	Taiwan

* China refers to mainland China.

### The leading journals

The top 20 resources accounted for 27.4% of the scientific output in the field studied, with nearly half published in ‘top five’ journals (Table 4). The Scopus database showed that *Evidence-Based Complementary and Alternative Medicines* ranked first in respect of citations (57), and surpassed PLOS Medicine (54), the *Chinese Journal of Integrative Medicine and Nicotine and Tobacco Research* (25 each). The journal with the most articles or most cited articles was *Evidence-Based Complementary and Alternative Medicines.*

**Table 4:** Output of articles in different journals

*Sources*	*Articles*	Location*	*H-index*	*Total citations*	*Year*
Evidence-Based Complementary and Alternative Medicines	6	UK	4	57	2013
Chinese Journal of Clinical Rehabilitation	4	China			2002
Journal of Traditional Chinese Medicine	4	China	1	3	2017
Chinese Journal of Integrative Medicine	3	China	3	25	2012
European Journal of Clinical Pharmacology	3	UK	2	21	2016
European Journal of Integrative Medicine	3	UK	2	5	2018
Chinese Journal of Six Lines of Diseases	3	China			2018
Mediterranean Medical Journal	2	Italy			2000
Chest	2	USA	2	11	2011
Explore: Journal of Science and Therapy	2	USA			2005
Comprehensive Cancer Treatment	2	USA			1974
International Journal of Urology	2	USA	2	11	2019
Journal of Clinical Rehabilitation Tissue Engineering Research	2	Japan			1997
Journal of Thoracic Diseases	2	Hong Kong			2009
Medicine (US)	2	USA	1	4	2018
Nicotine and Tobacco Research	2	UK	1	25	2012
PLOS Medicine	2	USA	2	54	2015
Singapore Medical Journal	2	Singapore	1	8	1980
World Journal of Pediatrics	2	China	2	8	2014
Chinese Acupuncture	2	China	1	3	2007

* China refers to mainland China.

### The leading journals, authors, publications

To analyze the leading scholars who contributed to the literature on TCM therapy for smoking cessation from 2005 to 2021, the number of published articles and the number of citations are presented in Table 5, in which the top 20 authors that contributed to the field (4.31 authors per paper, 1138 articles) are presented. Most papers (over 95%) were the product of co-authorship, and almost half were multi-author (48.1%). The number of single country publications (SCP) was 54 and the multiple country publications (MCP) was 26, the rate of MCP-Ratio was 18.58%, an indication of cooperation among scholars from different countries. In these countries, China and USA cooperated most frequently.

**Table 5:** Most productive authors on TCM therapy for smoking cessation

*Authors*	*Articles*	*H-index*	*TC*	*PY-start*	*City*
Wang Y	10	7	732	2011	Shanghai
Li Y	8	5	77	2015	Beijing
Li J	6	4	318	2014	Guangzhou
Li L	5	4	330	2015	Guangzhou
Li S	5	3	28	2015	Zhengzhou
Li X	5	2	136	2016	Beijing
Wang L	5	4	88	2015	Fuzhou
Wang Z	5	2	47	2012	Zhengzhou
Li Q	4	2	133	2019	Beijing
Wang C	4	3	38	2015	Beijing
Wang M	4	3	73	2015	Hong Kong
Wang Q	4	3	108	2018	Hangzhou
Zhang Q	4	3	303	2014	Zhengzhou
Chen J	3	3	54	2018	Beijing
Lu J	3	2	131	2019	Hangzhou
Tian Y	3	3	32	2015	Zhengzhou
Zhang Y	3	2	33	2018	Beijing
Chen L	2	3	258	2019	Beijing
Chen Y	2	1	5	2020	Shanghai
Lu W	1	1	9	2016	Beijing

Leading scholars were mostly from China (Table 5). The top 3 productive authors, Wang Y (10), Li Y (8) and Li J (6), were from Shanghai, Beijing and Guangzhou, respectively. The three cities are first-tier cities in China, where the level of economic development and financial strength are at the forefront. Concerning the authors’ impact, Wang Y ranked first both in the total citation index and H-Index^11^.

Finally, the research by top authors represented the breadth, quality, and other potential scholars from 2005 to 2021. The larger the circle area, the more output the author’s paper has. It revealed that there were only several authors who were interested in this topic in the beginning, but more and more authors participated over time, reaching the peak in 2020. The special emphasis was placed on their increasingly active role in promoting TCM treatment for smoking cessation.

### Leading publications

The analysis of the top articles on TCM therapy for smoking cessation was based on annual citations in the medical field (Supplementary file Table 1). The most cited articles were published in STROKE^12^, which was the most productive journal (the top total citations). Although the older papers were generally better known in the literature, the newer articles were more popular among the top five most cited papers. It confirmed that there was emerging research on TCM therapy for smoking cessation.

The main diseases caused by smoking in patients were stroke, diabetes, chest pains, and cancer^13^, while for healthy people, the diseases mainly included cardiovascular, cancer, diabetes, and hypertension. Smoking was also harmful to pregnant women and babies. Although there were many professional journals in STROKE (caused by cardiovascular disease), the most cited articles were published mainly in the fields involving Medicine –Clinical Neurology.

Moreover, highly cited papers published in the journal of *THORAX* covered respiratory system research topics. Most of these articles focused on patients’ Respiratory Asthma Guidelines such as Douglas (2008). Finally, highly cited papers published in the journal *LANCET ONCOL*, such as the one by Goss (2014).

### Three-field plots

In order to visualize the connections among scientific fields, we used three-field plots^14^, which simultaneously analyze the main relations from the selected fields (journals, authors, papers’ keywords and cited journals) and interpret how they were related, based on the method of Sankey diagram for three fields, which represents different flow diversion situations and the amount of traffic in a specific state. It displays related index through different colored rectangles proportional to the relationship value.

**Figure 2:**
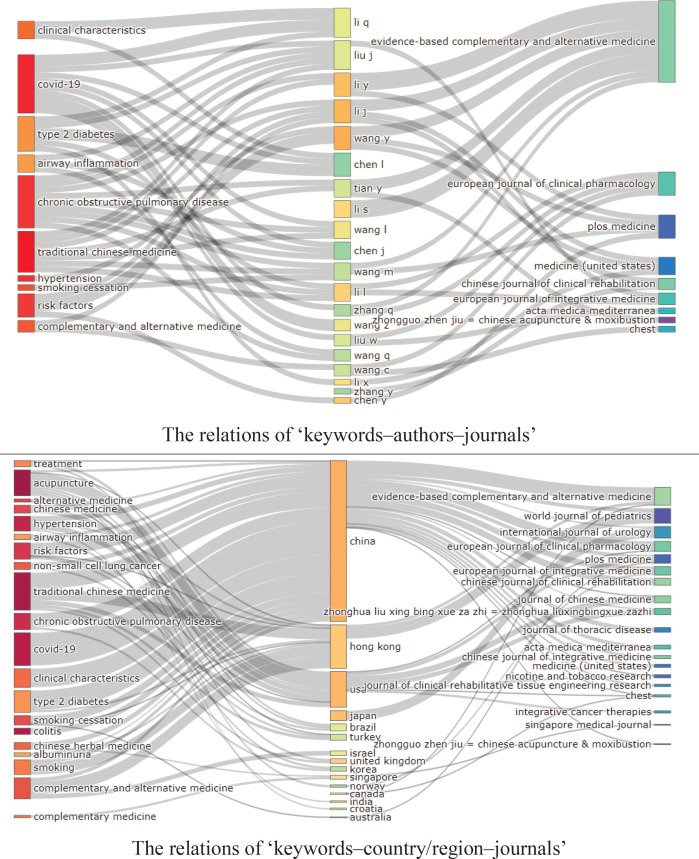
Three-field plots for ‘keywords–authors–journals’ and ‘keywords–country/region–journals’

The Sankey diagram was used to visualize data flow and volume among nodes. The wider the lines are, the more closely the nodes are linked. It revealed the relations of ‘keywords–authors–journals’ in representative scholars (Figure 2), by visualizing the relationship among paper keywords (left), authors (middle), and journals (right). The analysis revealed that, different scholars had paid different attention to the keywords about TCM therapy for smoking cessation such as COVID-19^15^, chronic^16^, type 2 diabetes^17^, and risk factor^18^. Besides, it showed that most of the scientific work was widely published in the journals: *Evidence-Based Complementary and Alternative Medicines, European Journal of Clinical Pharmacology, PLOS Medicine, and Chinese Journal of Clinical Rehabilitation.*

It revealed the relations of ‘keywords–country/region–journals’ in representative scholars (Figure 2), by visualizing the relationship among papers’ keywords (left), countries (middle) and cited journals (right). The analysis demonstrated the links among keywords, authors’ countries/areas and cited journals.

The two major journals with the most articles published by Chinese scholars were the *World Journal of Pediatrics and Evidence-Based Complementary and Alternative Medicine.* The major themes that Chinese scholars paid attention to were treatment methods such as traditional Chinese medicine (TCM), Chinese herbal medicine, and acupuncture. The related diseases concerned by scholars were mainly ‘hypertension’, ‘type 2 diabetes’ and ‘chronic obstructive pulmonary disease’. Some scholars^19^ believed that TCM for smoking cessation was a complementary and alternative medicine.

### Network analysis

Firstly, we analyzed the most common words and keywords during our research period, which allowed us to visualize the topics through word-cloud. Afterwards, we explored the treatment methods in healthy people and patients, respectively, via a tree-map and clustering analysis. Finally, we analyzed the 3C networks (co-occurrence; co-keywords; co-citation), and visualized a conceptual structure by a topic dendrogram.

### Word-cloud

**Figure 3:**
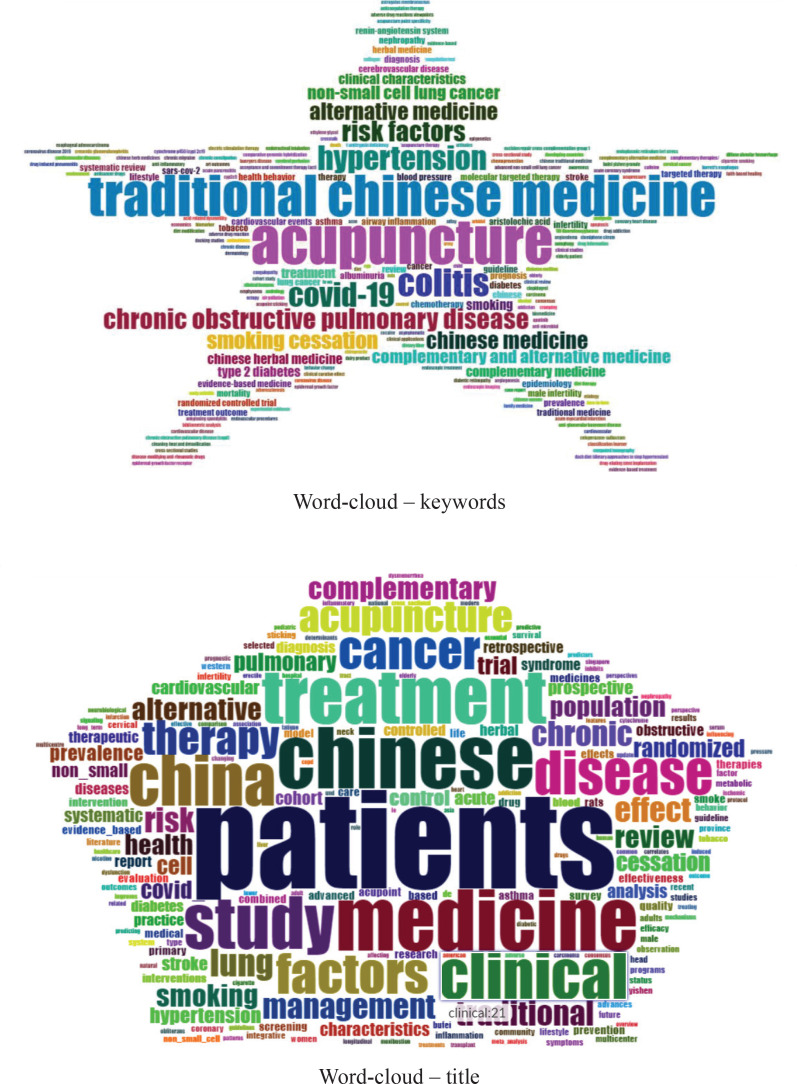
Word-clouds by keywords and by title

The most common words in the research literature were highlighted by the method of word-cloud, (Figure 3). It demonstrated the repetition frequency of words in the sample data. The more frequently the words were mentioned, the larger their sizes are in the selected sample.

The top keywords at the forefront in frequency such as ‘acupuncture’, ‘traditional Chinese medicine’, ‘colitis’, ‘hypertension’, ‘chronic obstructive pulmonary disease’, ‘risk factors’ and ‘alternative medicine’ were located in the center of map, largely and conspicuously. While for words mentioned in lower frequency such as ‘complementary medicine’, ‘treatment’, ‘type 2 diabetes’, ‘airway inflammation ‘ and ‘evidence-based medicine’, their sizes were smaller than the top words but still more prominent compared with the great majority.

The top keywords that occurred in titles were ‘patients’, ‘Chinese’, ‘medicine’, ‘treatment’, ‘clinical’, ‘disease’, ‘lung’, ‘cancer’, ‘therapy’, ‘acupuncture’, suggesting that acupuncture was one of the most commonly used words in titles. It illustrated that scholars mainly focus on smoking-related diseases therapies^20^. Besides, words such as ‘factors’, ‘effect’, ‘chronic’, ‘risk’, ‘management’, ‘alternative’, ‘complementary’, ‘traditional’, ‘randomized’, ‘trial’ and ‘control’ also recurred in many papers. These titles focused on the core content, test methods, and statistical methods.

The top keywords in abstracts according to the frequency they occurred in articles were ‘patients’, ‘treatment’, ‘therapy’, ‘clinical’, ‘disease’, ‘results’, located in the center of map. Besides, keywords such as ‘control’, ‘factors’, ‘acupuncture’, ‘cancer’, ‘blood’, ‘chronic’ and ‘hypertension’ also recurred in papers. This image demonstrates that the literature focused on the core concepts, patients, treatment methods, type of disease and results of TCM therapy on smoking cessation. Figure 3 illustrates the changing trend of hot topics in literature about TCM therapy for smoking cessation during the period 2005–2021. In 2020, new hotspots emerged in this field, which could be summarized by three keywords (COVID-19^21^, trial^22^, cell^23^). It revealed that in the post-epidemic era, scholars^24^ paid more attention to TCM therapy for smoking cessation.

### Tree-map – the healthy people

The tree-map explored the frequency and proportion of each word in the target topic, and illustrated the visual proportion in the size of the ‘rectangular’ area.

The diseases mentioned mainly cardiovascular, cancer, diabetes, and hypertension. The top words in the abstract were smokers (322), cardiovascular (191), cancer (175), diabetes (166), chronic (150), blood (141), pressure (96), hypertension (89), and pregnancy (84).

Treatment methods mainly included traditional Chinese medicine and acupuncture. The keywords were treatment (246), and acupuncture (100). People mentioned mainly participants (250), women (162), the public (158), and pregnancy (84). It revealed clearly that the top words were risk (500), intervention (232), control (219), evidence (141), clinical (105), significant (95) and random (80).

### Tree-map – the patients

Smoking is one of the risk factors for many diseases such as lung cancer, chronic bronchitis, and pulmonary fibrosis. The diseases of the patients concerned mainly cancer, diabetes, hematopathy, stroke, cardiovascular, diabetes, lung disease, carcinoma, hypertension and renal disease. The top words of the abstracts were cancer (49), diabetes (49), blood (45), stroke (33), cardiovascular (29), diabetic (29), lung (28), carcinoma (20), hypertension (20), renal (19) and colitis (8). Treatment methods mainly included traditional Chinese medicine and acupuncture. The top words at the forefront in frequency were traditional (31), herbal (23), and tongue (19).

The main types of people were smoker participants, the aged, women^25,26^, the public, adults, and pregnant, with the top word at the forefront in frequency being women (40). The research methods mainly included logistic regression models, meta-analysis methods, statistical significance tests, etc. The related top words were logistic (19), evidence (16), significant (31), and significantly (31).

### Co-keywords analysis

**Figure 4:**
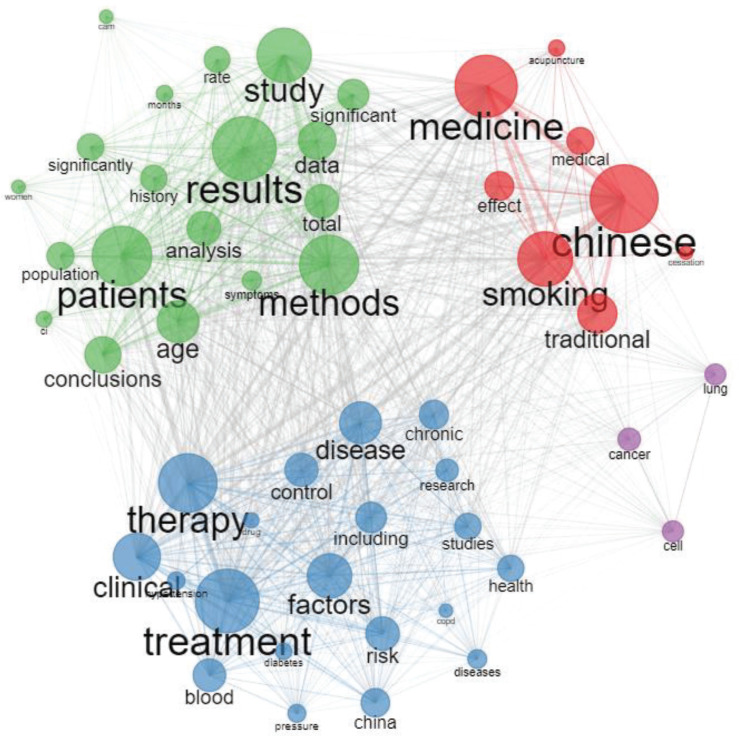
Co-occurrence network through the assessment of abstract terms

In order to present research hotspots on TCM therapy for smoking cessation, we performed a co-occurrence network analysis through abstracts (Figure 4). The link strength among keywords represented the number of publications by co-occurrence analysis. We could identify relationships among different concepts in the selected sample by the method of co-occurrence analysis. Therefore, we could observe what was the structure of a scientific article on a particular topic. In terms of link strength, the higher the association strength, the greater the similarity between terms.

The close interrelationships were highlighted among the topics by the co-occurrence network of abstracts. The treatment methods of TCM therapy for smoking cessation included treatment methods, drug therapy, controlled study, and treatment model. Many scholars explored the research from the perspective of a systematic review of evidence-based medicine, and the common topics of TCM therapy focused on the acupuncture, research method (method, control), type of disease (disease, blood, chronic, hypertension, diabetes), and evaluation (significantly, significant). To better present the type of research topics by linked keywords of the cluster analysis (especially the hierarchical clustering), it was used as follows. These articles were divided into three categories in selected samples: Cluster 1, with 7 words, including the methods and outcomes of the trial intervention; Cluster 2, with 13 words, including the clinical features and research methods; and Cluster 3, with 40 words, including types of diseases, TCM therapy.

The advantage of cluster analysis methods is that the existing research was mainly divided into several major themes distinctly. There were three types of analysis represented by the topic dendrogram. It revealed the flow of literature focused on TCM therapy for smoking cessation, clinical features (clinical), research method (randomized controlled trial, multi-center, double-blind), types of disease (stroke, lung, hypertension, diabetes, disease, hemopathy), and treatment methods (traditional Chinese medicine, acupuncture).

The co-occurrence network analysis was further explored to identify themes within a certain field, the relationships and the internal structure among these themes and their concentration. Therefore, we could present different topics by applying a clustering algorithm on the keyword network, which was finally displayed in the map.

In order to present the growth of topics and their relevance across the scientific field, the horizontal axis represented centrality and the vertical axis density. Based on this, four research quadrants in the field of TCM therapy for smoking cessation were drawn. Quadrant 1 (upper right corner) included a sports theme, both important and well-developed; and Quadrant 2 (upper left corner) included a very professional/niche subject which was well developed, but not for the current TCM therapy for the smoking cessation field; Quadrant 3 (lower left corner) included a new or a disappearing theme, which was a marginal topic, a poorly developed place, and might have just appeared or might be about to disappear; and Quadrant 4 (bottom right) included a basic theme, generally, a basic concept, revealing that the heart field of TCM therapy for smoking cessation was important but not well developed.

Therefore, as expected, the recurring cross-cutting themes (lower right quadrant) were ‘Chinese’, ‘traditional’, ‘medicine’, ‘patients’, ‘China’, ‘clinical’ and ‘risk’, ‘factor’, ‘population’, which were the most relevant to the area of knowledge considered. It demonstrated the methods of randomized trials related to TCM therapy for smoking cessation by the words ‘risk’ and ‘factor’, etc. The first cluster (lower right) focused on the treatment of TCM for smoking cessation according to their centrality and density. The second cluster (upper right), which was the ‘motor theme’, focused on the concepts on TCM therapy for smoking cessation. As for ‘therapy’, ‘acupuncture’, ‘smoking’, ‘study’, ‘cancer’ and ‘lung’ (upper left), which were linked to TCM therapy for smoking cessation (‘treatment’, ‘diagnosis’, ‘chronic’), developed well, but they were not important at present. Moreover, it is worth mentioning that ‘alternative’ and ‘complementary’ are still emerging themes on TCM therapy for smoking cessation (lower left quadrant).

### Collaboration network analysis

The influential authors and potential collaborations related to TCM therapy for smoking cessation were identified through a network analysis of publications, co-authors, and co-cited authors.

We could also have used the social network theory to find potential clusters of topics, clusters of researchers, and national collaborations among countries and regions. The size of the nodes is proportional to the number of papers published in the field in a certain country. The width of the lines is proportional to the number of collaboration articles. It was worth noting that the research on TCM therapy for smoking cessation mainly came from China (Supplementary file Figure 2). The wider the line between the two nodes, the closer the cooperation between the two authors in different institutions. The size of the node was proportional to the number of author’s articles. In addition, different colors indicated the year of the two authors’ collaborative papers. Besides, the different colors indicated the year of the collaborative paper between the two authors.

## DISCUSSION

As shown in the research literature, an increasing need for TCM therapy for smoking cessation has emerged. Our bibliometric analysis of related articles published from 2005 to 2021 demonstrated that the total number of articles per year had increased steadily during this period.

By analyzing keywords, the trends in a particular field revealed that the available literature mainly focused on the core concepts, patients, methods and results of TCM therapy for smoking cessation. Firstly, scholars focused on the mechanism and application fields of TCM therapy for smoking cessation; secondly, they used evidence-based medicine methods, such as meta-analysis, to systematically analyze the clinical effectiveness of TCM therapy for smoking cessation. To sum up, the articles focused mainly on the patients and treatment methods of TCM therapy for smoking cessation.

In the present study, more articles focused on the ways to quit smoking in Chinese medicine^27^. The network analysis based on the frequency of keywords revealed the trends was focused on TCM therapy, for smoking cessation, clinical features (clinical), research method (randomized controlled trial, multi-center, double-blind), types of disease (stroke, lung, hypertension, diabetes, disease, hemopathy), treatment methods (traditional Chinese medicine, acupuncture).

We identified two main types of studies (healthy people and patients) by a tree-map. The diseases of the patients concerned mainly cancer, diabetes, hematopathy, stroke, cardiovascular, diabetic, the lung disease, carcinoma, hypertension, and renal disease.

The main keywords used in the basic studies were risk (500), intervention (232), control (219), evidence (141), clinical (105), significant (95), and random (80). These analyses of keywords revealed that the diseases in healthy populations mainly included cardiovascular, cancer, diabetes, and hypertension.

In the current study, most of the diseases were related to cancer, diabetes and hematopathy, and specific diseases^28^ as mentioned by other scholars.

The systematic review^29^ articles mainly focused on the basic information of clinical research on traditional Chinese medicine, the randomized controlled trials of acupuncture for smoking cessation, the acupuncture stimulation techniques, and the efficacy and safety of acupuncture for smoking cessation^30^.

However, the bibliometric analysis provided the overall map of acupuncture and related interventions for smoking cessation^31^.

Additionally, we examined the trends in the co-occurrence network of abstracts, which emphasized the close interrelations among the topics covered in reviews, highlighting treatment methods including TCM therapy for smoking cessation, treatment methods, drug therapy, controlled study, and treatment model.

The keywords used in more recent articles were about the common types of TCM therapy, including acupuncture, research method (method, control), type of disease (disease, blood, chronic, hypertension, diabetes), and evaluation (significantly, significant).

The collaboration network analysis of countries and areas, organizations, and authors revealed that many research organizations in Asian countries produced high numbers of studies.

In addition to examining the top 10 countries and areas, organizations, and authors behind many academic articles, we identified China issued the most articles (96%). This revealed that Chinese scholars had played leading roles in TCM therapy for smoking cessation.

### Limitations

This study has some limitations. The bibliometric analysis of this study did not consider the content of the articles. In view of the development status of TCM therapies for smoking cessation, studies should focus on the improvement of evidence-based, evidence-driven and methodological quality on TCM therapy for smoking cessation, such as new treatment techniques (physical therapy) and development efforts. Furthermore, researchers should focus on outcome indicators of TCM therapy for smoking cessation, taking into account smoking craving, nicotine dependence, and daily smoking indicators such as changes in smoking volume, mood and physical symptoms.

## CONCLUSIONS

According to established search principles, abstracts, keywords and titles were chosen to reflect the main focus of the entire study. The main findings indicate that the most productive country in this field was China. The most productive institution was Beijing University. The most productive, cited journals were *Chinese Journal of Clinical Rehabilitation and Evidence-Based Complementary and Alternative Medicines.*

The dominant author that contributed to the research on TCM therapy for smoking cessation was Wang Y, who has the highest H-Index. Liu L, (2011, STROKE) had the highest centrality. Liu L (2011, STROKE) had the highest co-citation, citation number and centrality. It revealed that TCM therapy for smoking cessation was not recognized in a certain period of time as an auxiliary medical method.

Through three fields of Sankey diagram analysis, we could find that the authors paid attention to visualize the connections among the most important scientific fields. The two major journals with the most articles published by Chinese scholars were *World Journal of Pediatrics and Evidence-based Complementary and Alternative Medicine.* The two major themes that Chinese scholars paid most attention to were treatment methods such as traditional Chinese medicine, acupuncture, and COVID-19.

There were two main methods of TCM therapy for smoking cessation: traditional Chinese medicine and acupuncture. The diseases of healthy people concerned mainly cardiovascular, cancer, diabetes, and hypertension. The diseases of the patients concerned mainly cancer, diabetes, hemopathy, stroke, cardiovascular, diabetes, lung diseases, carcinoma, and hypertension. Treatment methods mainly included TCM and acupuncture. The research methods mainly included randomized controlled trials (RCT), double-blind and multi-center. It revealed that the hotspots and frontier trends of TCM therapy for smoking cessation were evaluated by the method of meta-analysis or systematic review.

The abstract of the literature focused on the core concepts, patients, methods, and results of TCM therapy for smoking cessation. The titles of the literature focused on the core content, test methods, and statistical methods of the main research on TCM therapy for smoking cessation. The keywords of the articles mainly focused on the patients and treatment methods of TCM therapy on smoking cessation.

## Data Availability

The data supporting this research are available from the authors on reasonable request.
