# Supplementary material for: Bibliometric analysis of traditional Chinese medicine for smoking cessation
Source: Tob Induc Dis. 2022 Nov 9;20:97. doi: 10.18332/tid/154961 (PMC9644233; doi:10.18332/tid/154961)
Supplement: Supplementary file 1 [file TID-20-97-s1.pdf]

Supplementary Table 1. Citation for different papers or journals

**Paper/Journal**	***DOI***	**Total Citations**	**TC***	**NTC****
LIU L, 2011, STROKE	*10.1161/STROKEAHA.111.635755*	592	49.33	4.67
DOUGLAS G, 2008, THORAX	*10.1136/thx.2008.097741*	443	29.53	3.97
GOSS PE, 2014, LANCET ONCOL	*10.1016/S1470-2045(14)70029-4*	282	31.33	6.60
LIU G, 2017, INT J MOL SCI	*10.3390/ijms18020367*	178	29.67	8.90
WHITTAKER P, 2004, LASERS MED SCI	*10.1007/s10103-004-0296-8*	163	8.58	3.24
LEUNG L, 2011, J AM BOARD FAM MED	*10.3122/jabfm.2011.04.100272*	157	13.08	1.24
WHITE AR, 2006, COCHRANE DATABASE SYST REV	*10.1002/14651858.CD000009.pub2*	141	8.29	4.57
MA GX, 1999, J COMMUNITY HEALTH	*10.1023/A:1018742505785*	139	5.79	1.00
JIA W, 2019, DIABETES METAB RES REV	*10.1002/dmrr.3158*	128	32.00	7.59
HOFFMANN JC, 2008, Z GASTROENTEROL	*10.1055/s-2008-1027796*	120	8.00	1.07

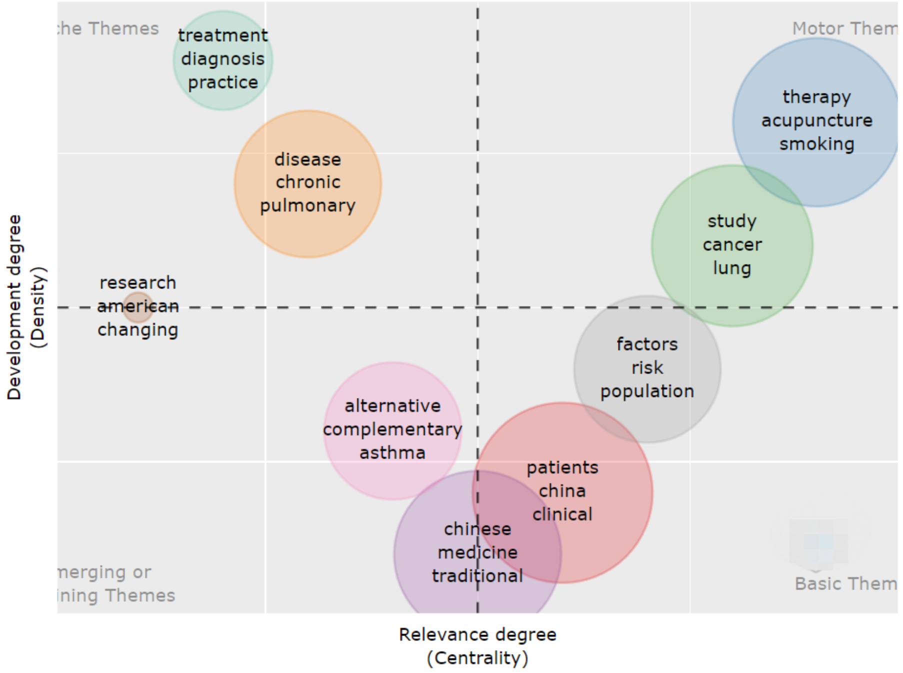

Supplementary Figure 1. Thematic map of titles

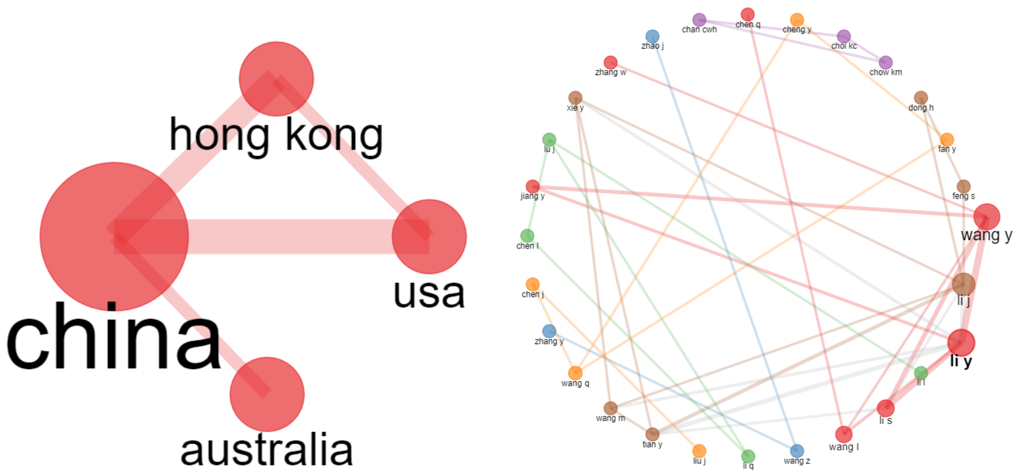

Supplementary Figure 2: Collaboration Network, Region & Author

© 2022 Xing J.
